# Supplementary material for: Active site specificity profiling datasets of matrix metalloproteinases (MMPs) 1, 2, 3, 7, 8, 9, 12, 13 and 14
Source: Data Brief. 2016 Feb 22;7:299–310. doi: 10.1016/j.dib.2016.02.036 (PMC4777984; doi:10.1016/j.dib.2016.02.036)
Supplement: Supplementary file 10 — Supplementary material [file mmc10.zip › WebPICS_hMMP13_T_1%/P2prime.html]

 

PICS results


|  |  |
| --- | --- |
| **P2prime\_H**  8 in 143 sites   5.6 %    effects > 10 perc. pnts.  (vice-versa in brackets)  P1\_D: 17.3 (12.6) |  |
| **P2prime\_I**  16 in 143 sites   11.2 %    effects > 10 perc. pnts.  (vice-versa in brackets)  P1\_Q: 20.8 (55.5)   P1prime\_V: 13.8 (13.8)   P3prime\_N: 19.4 (38.8) |  |
| **P2prime\_K**  14 in 143 sites   9.8 %    effects > 10 perc. pnts.  (vice-versa in brackets)  P2\_A: 24.0 (12.4)   P1\_G: 31.8 (17.1)   P1prime\_W: 29.4 (45.8)   P3prime\_D: 11.6 (11.6) |  |
| **P2prime\_L**  14 in 143 sites   9.8 %    effects > 10 perc. pnts.  (vice-versa in brackets)  P1\_S: 12.3 (13.3)   P3prime\_A: 22.6 (10.9) |  |
| **P2prime\_M**  4 in 143 sites   2.8 %    effects > 10 perc. pnts.  (vice-versa in brackets)  P2\_M: 47.2 (47.2) |  |
| **P2prime\_Q**  12 in 143 sites   8.4 %    effects > 10 perc. pnts.  (vice-versa in brackets)  P2\_H: 13.9 (41.6)   P1\_H: 13.2 (31.6)   P1\_P: 23.5 (20.2)   P1prime\_Q: 27.0 (36.0)   P3prime\_H: 25.6 (28.0) |  |
| **P2prime\_T**  8 in 143 sites   5.6 %    effects > 10 perc. pnts.  (vice-versa in brackets)  P1\_P: 27.7 (15.8)   P1prime\_C: 19.4 (19.4)   P3prime\_S: 40.9 (25.2) |  |
| **P2prime\_V**  21 in 143 sites   14.7 %    effects > 10 perc. pnts.  (vice-versa in brackets)  P2\_S: 10.5 (11.6)   P1\_N: 18.1 (25.3) |  |
| **P2prime\_W**  4 in 143 sites   2.8 %    effects > 10 perc. pnts.  (vice-versa in brackets)  P1prime\_M: 45.8 (30.5)   P3prime\_S: 40.9 (12.6) |  |
